# Supplementary material for: Reconfigurable spin current transmission and magnon–magnon coupling in hybrid ferrimagnetic insulators
Source: Nat Commun. 2024 Mar 12;15:2234. doi: 10.1038/s41467-024-46330-8 (PMC10933317; doi:10.1038/s41467-024-46330-8)
Supplement: Supplementary file 1 — Supplementary Information [file 41467_2024_46330_MOESM1_ESM.pdf]

SUPPLEMENTARY INFORMATION

Reconfigurable spin current transmission and magnon-magnon coupling in hybrid ferrimagnetic insulators

Yan Li[1], Zhitao Zhang[2], Chen Liu[1], Dongxing Zheng[1], Bin Fang[1], Chenhui Zhang[1], Aitian Chen[1], Yinchang Ma[1], Chunmei Wang[2], Haoliang Liu[2*], Ka Shen[3*], Aurélien Manchon[4], John Q. Xiao[5], Ziqiang Qiu[6], Can-Ming Hu[7], and Xixiang Zhang[1*]

[1]Physical Science and Engineering Division, King Abdullah University of Science and Technology (KAUST), Thuwal 23955–6900, Saudi Arabia

[2]Guangdong Provincial Key Laboratory of Semiconductor, Optoelectronic Materials and Intelligent Photonic Systems, School of Science, Harbin Institute of Technology (Shenzhen), Shenzhen 518055, China

[3]The Center for Advanced Quantum Studies and Department of Physics, Beijing Normal University, Beijing 100875, China

[4]Aix-Marseille Univ, CNRS, CINaM, Marseille, France

[5]Department of Physics and Astronomy, University of Delaware, Newark, Delaware 19716, USA

[6]Department of Physics, University of California at Berkeley, Berkeley, CA 94720, USA

[7]Department of Physics and Astronomy, University of Manitoba, Winnipeg, Canada R3T 2N2

[*]Corresponding author: Xixiang Zhang (email: xixiang.zhang@kaust.edu.sa), Haoliang Liu (email: liuhaoliang@hit.edu.cn) and Ka Shen (email: kashen@bnu.edu.cn)

Supplementary Note 1: Calculation of magnetic configuration and the low frequency dynamics

In order to describe the ground state spin configuration and the low frequency dynamics, we employ a minimal model consisting of the exchange between macrospins and their responses to an external magnetic field as

$$H = -\mu_B\left(|g_{Fe}|\boldsymbol{S}_{Fe}^{YIG} + |g_{Fe}|\boldsymbol{S}_{Fe}^{GdIG} + |g_{Gd}|S_{Fe}^{GdIG}\right)\cdot\boldsymbol{B} + A_{Fe-Fe}\boldsymbol{S}_{Fe}^{GdIG}\cdot\boldsymbol{S}_{Fe}^{YIG} + A_{Fe-Gd}\boldsymbol{S}_{Gd}^{GdIG}\cdot\boldsymbol{S}_{Fe}^{GdIG}. \quad \text{(S1)}$$

Here, the macrospins are defined as $S_{Fe}^{YIG(GdIG)} = 8S_{Fe}N_{YIG(GdIG)}$ and $S_{Gd}^{YIG(GdIG)} = 24S_{Gd}N_{GdIG}$ with $N_{YIG(GdIG)}$ being the number of cubic unit cells in the two layers, $S_{Fe(Gd)}$ the spin of single ion and $g_{Fe(Gd)}$ the g-factor. The interaction parameter between Fe and Gd in GdIG can be expressed by $A_{Fe-Gd} = (4J_{ac} - 2J_{dc})/(8N_{GdIG})$ with J_{ac} and J_{dc} being the exchange parameters between a Gd ion with its a- and c-site Fe neighbors[1-3]. The coupling strength between the two layers A_{Fe-Fe} is treated as an adjustable parameter for a better comparison with the observations. Defining $\theta_{Y(G)}$ as the canting angle of $S_{Fe}^{YIG(GdIG)}$ with respect to the z axis (along the magnetic field) and assuming an antiparallel configuration of Gd and Fe spins in the GdIG layer in both ground state and low frequency dynamics, the measured magnetization in the field direction thus can be expressed as $m_z = |g_{Fe}|\mu_B S_{Fe}^{YIG}\left[cos\theta_Y + (N_{GdIG}/N_{YIG})\left(1 - 3\left|g_{Gd}\,S_{Gd}^{GdIG}/g_{Fe}\,S_{Fe}^{GdIG}\right|\right)cos\theta_G\right]$. Considering temperatures far below the Curie temperature, we take $S_{Fe-Fe}^{YIG(GdIG)}$ as a constant and use Brillouin function to capture the strong temperature dependence of the Gd spins, i.e., $S_{Gd}^{GdIG}(T)/S_{Gd}^{GdIG}(0K) \simeq Bs(g\mu_B\,B_{ex}/k_B\,T)$, where the effective field due to Fe-Gd interaction is adopted to be $B_{ex} = 23$ T to match the compensation temperature (~200K) of the GdIG layer. The canting angles thus can be obtained by minimizing the total energy $E = -Bm_z + A_{Fe-Fe}S_{Fe}^{GdIG}S_{Fe}^{YIG}cos(\theta_Y - \theta_G)$. By further performing Holstein-Primakoff transformation in the local equilibrium spin coordinate systems[4], we derive an effective Hamiltonian for spin excitation as

$$H_m = A_1 a_{Fe'}^{\dagger}a_{Fe'} + A_2 a_{Fe}^{\dagger}a_{Fe} + A_3 a_{Gd}^{\dagger}a_{Gd} + (D_1 a_{Fe}^{\dagger}a_{Fe'} + D_2 a_{Fe}^{\dagger}a_{Fe'}^{\dagger} + D_3 a_{Fe}^{\dagger}a_{Gd}^{\dagger} + h.c.)\,. \quad \text{(S2)}$$

Here, $a_{Fe'}$, a_{Fe}, and a_{Gd} are annihilation operators of spin excitation for S_{Fe}^{YIG}, S_{Fe}^{GdIG}, and S_{Gd}^{GdIG}, respectively. The coefficients are defined as

$$A_1/\hbar = \omega_B cos\theta_Y + \omega_c cos(\theta_Y - \theta_G), \quad \text{(S3)}$$

$$A_2/\hbar = \omega_B cos\theta_G + \omega_c(N_{YIG}/N_{GdIG})cos(\theta_Y - \theta_G) + \omega_{ex}(3\,S_{Gd}/S_{Fe}), \quad \text{(S4)}$$

$$A_3/\hbar = \omega_{ex} - \omega_B(g_{Gd}/g_{Fe})cos\theta_G, \quad \text{(S5)}$$

$$C_1/\hbar = -(\omega_c/2)[1 + cos(\theta_Y - \theta_G)]\sqrt{N_{YIG}/N_{GdIG}}, \quad \text{(S6)}$$

$$C_2/\hbar = (\omega_c/2)[1 - cos(\theta_Y - \theta_G)]\sqrt{N_{YIG}/N_{GdIG}}, \quad \text{(S7)}$$

$$C_3/\hbar = -\omega_{ex}\sqrt{3\,S_{Gd}/S_{Fe}}\ . \quad \text{(S8)}$$

Besides, $\hbar\omega_B = |g_{Fe}|\mu_B B$, $\hbar\omega_{ex} = (4J_{ac} - 2J_{dc})S_{Fe}$, and $\hbar\omega_c = -8N_{GdIG}A_{Fe-Fe}S_{Fe}$. The diagonalization procedure leads to i-th magnon operator $a_i = \sum_{j=Fe',Fe,Gd}(\eta_{i,j}a_j + \zeta_{i,j}a_j^\dagger)$ and the amplitude of spin excitation in the YIG layer is given by $\psi_{YIG}^S = |\eta_{i,Fe}|^2 - |\zeta_{i,Fe}|^2$. In numerical calculation, we take $\omega_{ex} = 521$ GHz and $\omega_c = 0.39$ GHz.

The hysteresis loops calculated at various representative temperatures are presented in Figs. 1e-g in the main text. The dependence of θ_G and θ_Y on the applied magnetic field on the applied magnetic field is demonstrated in Supplementary Figs. 1a-c.

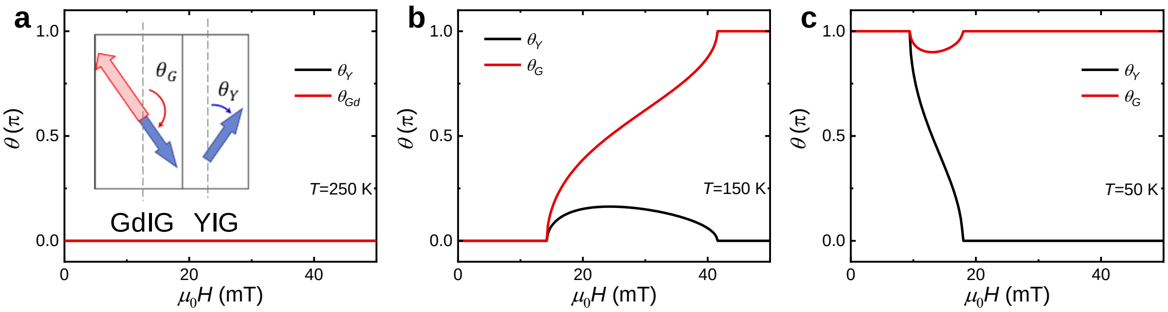

Supplementary Fig. 1| Evolution of magnetic configurations as a function of the applied in-plane magnetic field at various temperatures. a-c, Calculated angles θ_G and θ_Y as functions of the applied magnetic field at 250 K, 150 K, and 50 K, respectively. The inset in **a** is the definition of θ_G and θ_Y.

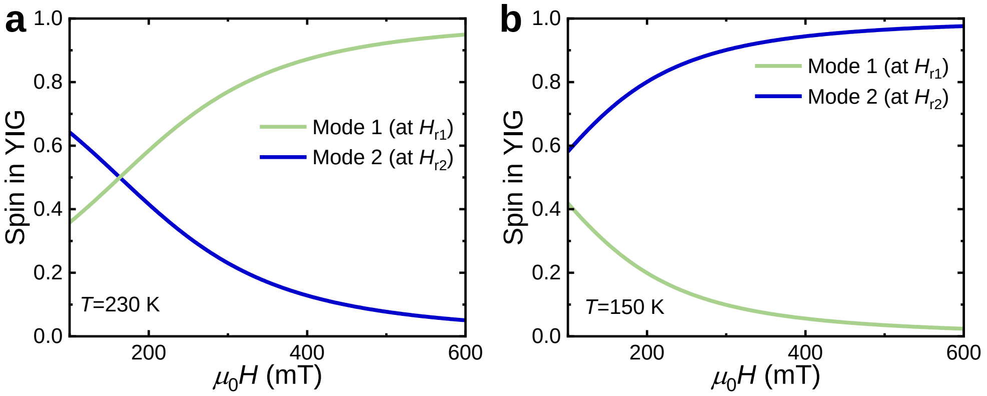

Supplementary Fig. 2| Calculation of reconfigurable coherent magnon transmission. a-b, Theoretical amplitudes of spin excitation contributed by the two coupled modes at the Pt/YIG interface as functions of the applied magnetic field at T=230 K and 150 K, respectively.

To describe magnon-magnon coupling, we can express the microscopic spin model using creation ($\hat{a}^\dagger$ and $\hat{b}^\dagger$) and annihilation ($\hat{a}$ and $\hat{b}$) operators:

$$H = \hbar\omega_{0a}\left(\hat{a}^\dagger\hat{a} + \frac{1}{2}\right) + \hbar\omega_{0b}\left(\hat{b}^\dagger\hat{b} + \frac{1}{2}\right) + \hbar\left(g\hat{a}\hat{b}^\dagger + g^*\hat{a}^\dagger\hat{b}\right) \quad \text{(S9)}$$

Here, ω_{0a} and ω_{0b} are the uncoupled magnon modes. g is the coupling strength.

Supplementary Note 2: Coupled LLG equations with coherent spin pumping term

For a system with two coupled magnetizations, the magnetization precession can be described via two expanded Landau-Lifshitz-Gilbert (LLG) equations,

$$\frac{d\mathbf{M}_1}{dt} = -\mu_0\gamma_1\mathbf{M}_1 \times (\mathbf{H}_1^{eff} + A_{12}^{eff}\mathbf{M}_2\,) + \frac{\alpha_1}{M_1}\mathbf{M}_i \times \frac{d\mathbf{M}_1}{dt} + \frac{\Delta\alpha_1^{SP}}{M_1}\mathbf{M}_1 \times \frac{d\mathbf{M}_1}{dt} - \frac{\Delta\alpha_{12}^{SP}}{M_2}\mathbf{M}_2 \times \frac{d\mathbf{M}_2}{dt} \quad \text{(S10)}$$

$$\frac{d\mathbf{M}_2}{dt} = -\mu_0\gamma_2\mathbf{M}_2 \times (\mathbf{H}_2^{eff} + A_{21}^{eff}\mathbf{M}_1\,) + \frac{\alpha_2}{M_2}\mathbf{M}_2 \times \frac{d\mathbf{M}_2}{dt} + \frac{\Delta\alpha_2^{SP}}{M_2}\mathbf{M}_2 \times \frac{d\mathbf{M}_2}{dt} - \frac{\Delta\alpha_{21}^{SP}}{M_1}\mathbf{M}_1 \times \frac{d\mathbf{M}_1}{dt} \quad \text{(S11)}$$

Here, γ_i , α_i , and M_i are the gyromagnetic ratio, Gilbert damping constant, saturation magnetization of the i-th layer (i=1, 2), respectively. $\mathbf{H}_{eff i}$ is the effective field in the i-th layer including the external field, anisotropy field and demagnetizing field. $A_{12} = \frac{J_{eff}}{\mu_0 M_1 M_2 t_1}$ and $A_{21} = \frac{J_{eff}}{\mu_0 M_1 M_2 t_2}$ are the effective exchange interaction with the effective exchange coupling constant J_{eff}

between two layers. $\Delta\alpha_i^{SP}$ and $\Delta\alpha_{ij}^{SP}$ are enhanced damping due to coherent spin pumping with[5,6] $\Delta\alpha_{ij}^{SP} = \frac{\Delta\alpha_i^{SP} M_i}{M_j}$.

As the magnetization is precessing around the z-axis with a small precession angle, the magnetization is written as,

$$\mathbf{M_1} = \{m_{1x}e^{i\omega t}, m_{1y}e^{i\omega t}, \mu_0 M_1\}, \quad \text{(S12)}$$

$$\mathbf{M_2} = \{m_{2x}e^{i\omega t}, m_{2y}e^{i\omega t}, \mu_0 M_2\}. \quad \text{(S13)}$$

The effective field $\mathbf{H}_{eff\,i}$ of the *i*-layer, including the microwave field and effective uniaxial anisotropy field, is written as,

$$\mathbf{H}_{eff1} = \{h_{rf}e^{i\omega t}, -\mu_0 m_{1y}e^{i\omega t}, \mu_0(H_{ext} + H_{u1})\}, \quad \text{(S14)}$$

$$\mathbf{H}_{eff2} = \{h_{rf}e^{i\omega t}, -\mu_0 m_{2y}e^{i\omega t}, \mu_0(H_{ext} + H_{u2})\}. \quad \text{(S15)}$$

The coupled LLG equations can be translated into the following matrix equation:

$$D \cdot \begin{bmatrix} m_{1x} \\ m_{1y} \\ m_{2x} \\ m_{2y} \end{bmatrix} = \begin{bmatrix} 0 & 0 & 0 & 0 \\ -\mu_0 M_{1s} & 0 & 0 & 0 \\ 0 & 0 & 0 & 0 \\ 0 & 0 & -\mu_0 M_{2s} & 0 \end{bmatrix} \cdot \begin{bmatrix} h_{rf} \\ 0 \\ h_{rf} \\ 0 \end{bmatrix}. \quad \text{(S16)}$$

Here, the matrix D is defined as $D = D_m^a + D_m^b + \mathrm{g}_1 + \mathrm{g}_2$.

$$D_m^a = \begin{bmatrix} \frac{i\omega}{\gamma_1} & \mu_0(H_{ext} + M_1 + H_{u1}) & 0 & 0 \\ -\mu_0(H_{ext} + H_{u1}) & \frac{i\omega}{\gamma_1} & 0 & 0 \\ 0 & 0 & \frac{i\omega}{\gamma_2} & \mu_0(H_{ext} + M_2 + H_{u2}) \\ 0 & 0 & -\mu_0(H_{ext} + H_{u2}) & \frac{i\omega}{\gamma_2} \end{bmatrix}; \text{(S17)}$$

$$D_m^b = \begin{bmatrix} 0 & \mu_0 A_{12} M_2 & 0 & -\mu_0 A_{12} M_1 \\ -\mu_0 A_{12} M_2 & 0 & \mu_0 A_{12} M_1 & 0 \\ 0 & -\mu_0 A_{21} M_2 & 0 & \mu_0 A_{21} M_1 \\ \mu_0 A_{21} M_2 & 0 & -\mu_0 A_{21} M_1 & 0 \end{bmatrix}; \quad \text{(S18)}$$

$$
g_1 = \begin{bmatrix} 0 & \frac{i\alpha_1\omega}{\gamma_1} & 0 & 0 \\ -\frac{i\alpha_1\omega}{\gamma_1} & 0 & 0 & 0 \\ 0 & 0 & 0 & \frac{i\alpha_2\omega}{\gamma_2} \\ 0 & 0 & -\frac{i\alpha_2\omega}{\gamma_2} & 0 \end{bmatrix}; \tag{S19}
$$

$$
g_2 = \begin{bmatrix} 0 & \frac{i\Delta\alpha_1^{SP}\omega}{\gamma_1} & 0 & -\frac{i\Delta\alpha_{12}^{SP}\omega}{\gamma_1} \\ -\frac{i\Delta\alpha_1^{SP}\omega}{\gamma_1} & 0 & \frac{i\Delta\alpha_{12}^{SP}\omega}{\gamma_1} & 0 \\ 0 & -\frac{i\Delta\alpha_{21}^{SP}\omega}{\gamma_2} & 0 & \frac{i\Delta\alpha_2^{SP}\omega}{\gamma_2} \\ \frac{i\Delta\alpha_{21}^{SP}\omega}{\gamma_2} & 0 & -\frac{i\Delta\alpha_2^{SP}\omega}{\gamma_2} & 0 \end{bmatrix}. \tag{S20}
$$

As the above expressions contain completely all effects of the relevant coupling in the bilayer dynamics, it is more convenient to project them into the Kittel modes of the two layers, whose frequencies are given by $D = D_m^a + D_m^b + g_1 + g_2$. This can be done by performing a unitary transformation,

$$
(T^{-1}DT)\cdot T^{-1}\begin{bmatrix} m_{1x} \\ m_{1y} \\ m_{2x} \\ m_{2y} \end{bmatrix} = T^{-1}\begin{bmatrix} 0 & 0 & 0 & 0 \\ -\mu_0 M_1 & 0 & 0 & 0 \\ 0 & 0 & 0 & 0 \\ 0 & 0 & -\mu_0 M_2 & 0 \end{bmatrix} T\cdot T^{-1}\begin{bmatrix} h_{rf} \\ 0 \\ h_{rf} \\ 0 \end{bmatrix}
$$

and taking the upper left 2×2 diagonal block in $T^{-1}DT$ with the first two basis in, $T^{-1}\cdot [m_{1x}, m_{1y}, m_{2x}, m_{2y}]^T$. Here, the transformation matrix is defined as $T = \begin{bmatrix} 1 & 0 & 1 & 0 \\ -i\nu_1 & 0 & i\nu_1 & 0 \\ 0 & 1 & 0 & 1 \\ 0 & -i\nu_2 & 0 & i\nu_2 \end{bmatrix}$ with $\nu_i = \sqrt{(H_{ext}+H_{ui})/(H_{ext}+M_i+H_{ui})}$.

The reduced LLG equations are then expressed as

$$
\tilde{E}\cdot\begin{bmatrix} m_{1x} + \frac{i}{\nu_1} m_{1y} \\ m_{2y} + \frac{i}{\nu_2} m_{2y} \end{bmatrix} = \begin{bmatrix} -\mu_0 M_1 \frac{1}{\nu_1} h_{rf} \\ -\mu_0 M_2 \frac{1}{\nu_2} h_{rf} \end{bmatrix}. \tag{S21}
$$

Here, the eigenfrequency of Kittel modes $\pm\omega_i = \pm\gamma_i\mu_0\sqrt{(H_{ext}+H_{ui})/(H_{ext}+M_i+H_{ui})}$ are obtained from the diagonalization of D_m^a, and

$$\tilde{E} = \begin{bmatrix} \frac{\omega-\omega_1}{\gamma_1} & 0 \\ 0 & \frac{\omega-\omega_2}{\gamma_2} \end{bmatrix} +$$

$$\begin{bmatrix} -\frac{1}{2}\left(\nu_1 + \frac{1}{\nu_1}\right)\left(\mu_0 A_{12} M_2 + i\frac{\alpha_1+\Delta\alpha_1^{SP}}{\gamma_1}\omega\right) & \frac{1}{2}\left(\nu_2 + \frac{1}{\nu_1}\right)\left(\mu_0 A_{12} M_1 + i\frac{\Delta\alpha_{12}^{SP}}{\gamma_1}\omega\right) \\ \frac{1}{2}\left(\nu_1 + \frac{1}{\nu_2}\right)\left(\mu_0 A_{21} M_2 + i\frac{\Delta\alpha_{21}^{SP}}{\gamma_2}\omega\right) & -\frac{1}{2}\left(\nu_2 + \frac{1}{\nu_2}\right)\left(\mu_0 A_{21} M_1 + i\frac{\alpha_2+\Delta\alpha_2^{SP}}{\gamma_2}\omega\right) \end{bmatrix}.$$

The magnetic susceptibility of the two layers can be calculated by $\begin{bmatrix} \chi_{x_1x_1} \\ \chi_{x_1y_1} \\ \chi_{x_1x_2} \\ \chi_{x_1y_2} \end{bmatrix} = D^{-1}\begin{bmatrix} 0 \\ -\mu_0 M_1 \\ 0 \\ 0 \end{bmatrix}$ and $\begin{bmatrix} \chi_{x_2x_1} \\ \chi_{x_2y_1} \\ \chi_{x_2x_2} \\ \chi_{x_2y_2} \end{bmatrix} = D^{-1}\begin{bmatrix} 0 \\ 0 \\ 0 \\ -\mu_0 M_2 \end{bmatrix}$. The total magnetic susceptibility can be written as:

$$\chi_{total} = \chi_{x_1x_1} + \chi_{x_1x_2} + \chi_{x_2x_1} + \chi_{x_2x_2}. \tag{S22}$$

According to Eq. (S22), the FMR spectrum at 200 K and 150 K were calculated, as shown in Supplementary Fig. 3. The calculated spectra reproduce the magnon-magnon coupling [see Supplementary Figs. 3c and f] and the redistribution of precession energy in hybrid systems, and also elucidates the difficulty in clearly detecting peak 1 due to the small magnetization and large damping of GdIG around $T_{M,GdIG}$ ~210 K [see Supplementary Figs. 3b and e].

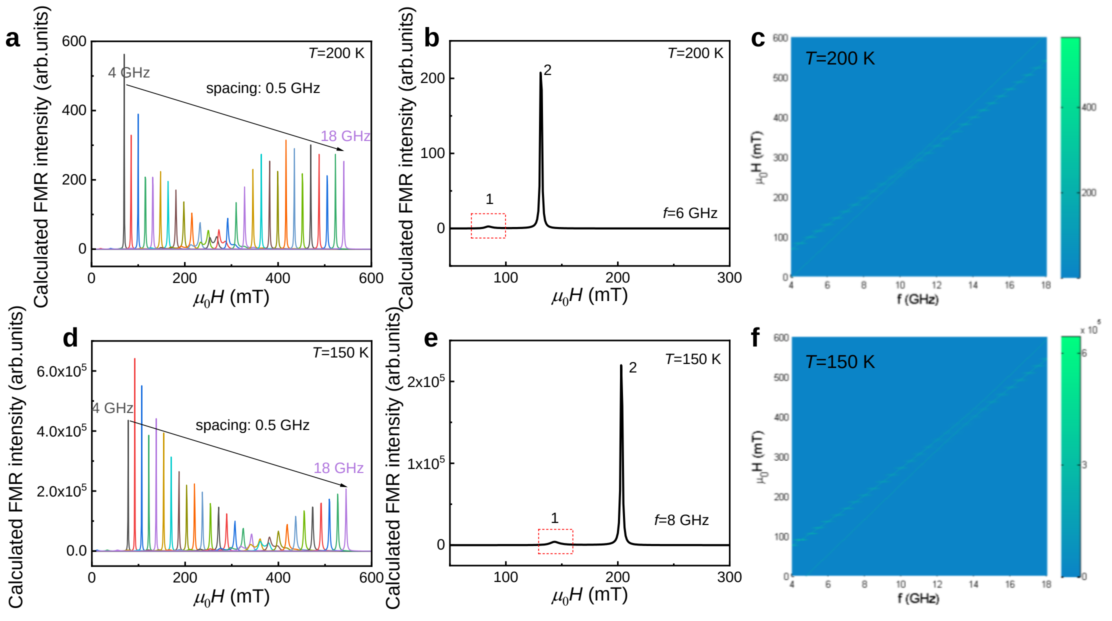

Supplementary Fig. 3 Calculated FMR spectra at 200 K and 150 K. a, Calculated FMR peaks at various frequencies at *T*=200 K. **b,** Calculated FMR absorption as a function of magnetic field at *f*=6 GHz and *T*=200 K. **c,** FMR spectrum based on the calculated FMR data

presented in **a**. The solid lines represent the peak positions. **d,** Calculated FMR peaks at various frequencies at T=150 K. **e,** Calculated FMR absorption as a function of magnetic field at f=8 GHz and T=150 K. **f,** FMR spectrum based on the calculated FMR data presented in **d**. The solid lines represent the peak positions.

Supplementary Note 3: Sample characterization

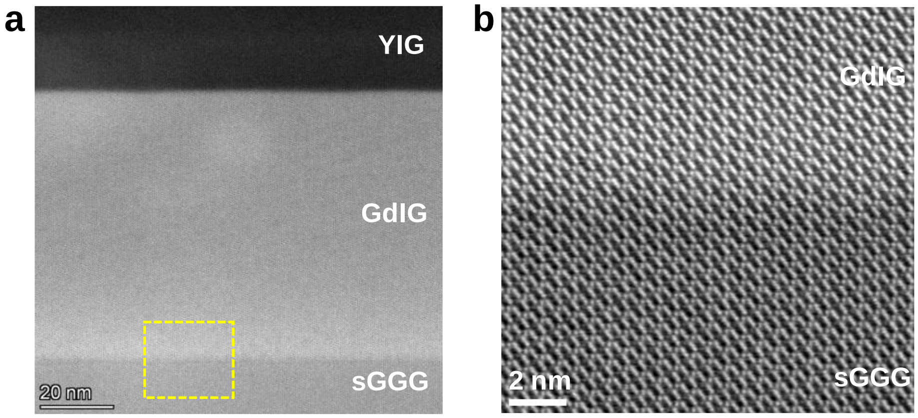

Supplementary Fig. 4| Structure of sGGG/GdIG/YIG. **a**, Cross-sectional STEM image including the sGGG/GdIG and GdIG/YIG interfaces. **b**, Atomic-resolution STEM image of the GdIG/YIG interface.

Supplementary Note 4: FMR and spin pumping-induced ISHE experiments

The FMR spectral lines are fitted using the superposition of Lorentzian and anti-Lorentzian functions,

$$\frac{dI}{dH} = S_0 + S_1 \frac{\left(\frac{\Delta H}{2}\right)(H-H_r)}{[(H-H_r)^2+(\frac{\Delta H}{2})^2]^2} + S_2 \frac{\left(\frac{\Delta H}{2}\right)-(H-H_r)^2}{[(H-H_r)^2+(\frac{\Delta H}{2})^2]^2} \quad \text{(S21)}$$

where H_r, ΔH and S_0, are resonance field, resonance linewidth, offset, respectively. S_1 and S_2 are the coefficient of the Lorentz and anti-Lorentz line terms.

Supplementary Figs. 5a-f display the FMR measurements above $T_{M,\mathrm{GdIG}}$. Two resonance modes were clearly identified in Supplementary Figs. 5a and d. The observed H_r versus f exhibit two well-separated branches at 300 K and 225 K, as shown in Supplementary Figs. 5b and e. ΔH scales linearly with f for both modes, as shown in Supplementary Figs. 5c and f.

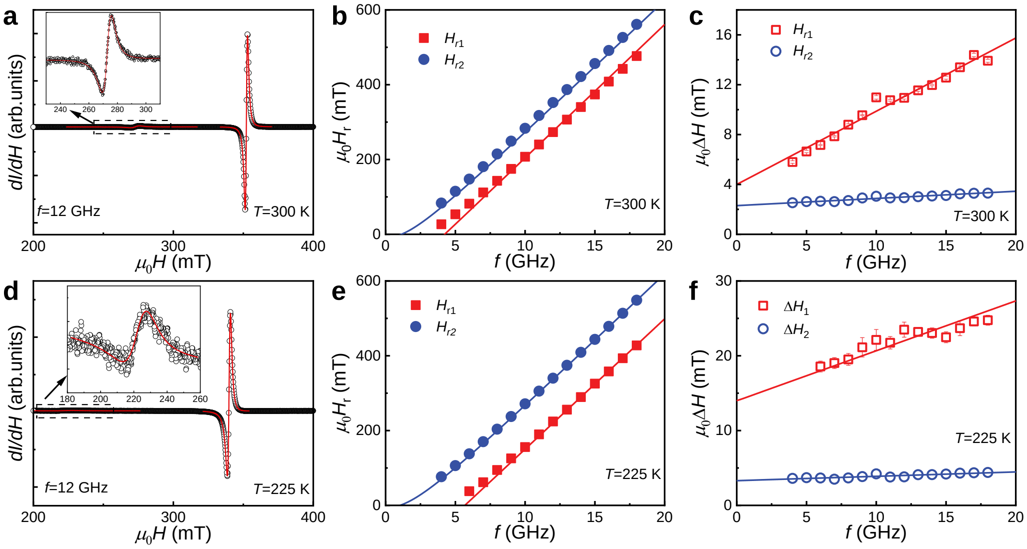

Supplementary Fig. 5| FMR measurements above $T_{M,\mathrm{GdIG}}$. **a**, Derivative FMR absorption spectra measured at T=300 K and f=12 GHz. **b-c**, Resonant field H_r and resonant linewidth ΔH extracted from the FMR spectra plotted against the resonant frequency f at T=300 K. **d**, Derivative FMR absorption spectra measured at T=225 K and f=12 GHz. **e-f**, Resonant field H_r and resonant linewidth ΔH extracted from the FMR spectra plotted against the resonant frequency f at T=225 K. Error bars represent fitting uncertainty.

Supplementary Figs. 6a-b displays the fits of H_r versus f and ΔH versus f at 150 K over a wider frequency range. It can be observed that the fitting curve of ΔH shows a tendency towards a linear relationship as the frequency increase.

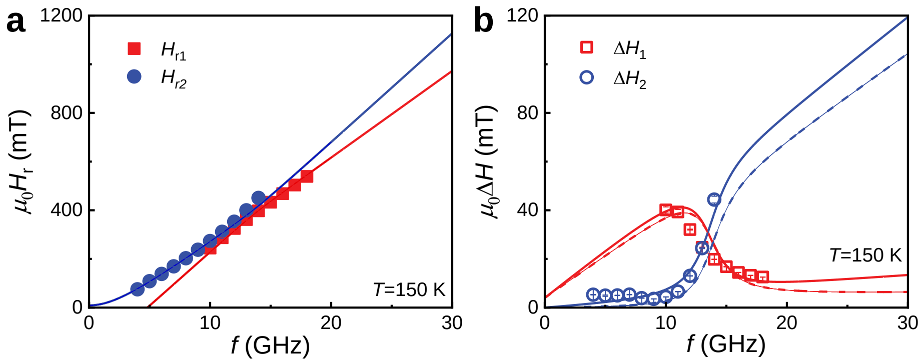

Supplementary Fig. 6| Fits of H_r versus f and ΔH versus f at 150 K over a wider frequency range. **a**, Frequency dependence of the resonant magnetic field H_r . **b**, Frequency dependence of the resonant linewidth ΔH. The solid (dash) lines show the fitting results with (without) coherent spin pumping.

Three-magnon processes were also assessed. If a parabolic magnon dispersion ($f = f_{FMR} + Ak^2$) is considered, the frequency f_{FMR}, corresponding to the FMR mode, is already the minimum value of frequency in the magnon dispersion, making it impossible to further split into two magnons. A more realistic magnon dispersion with magnetic dipole-dipole interactions, allows for the occurrence of three-magnon processes. In this case, the FMR frequency is approximately given by $f_{FMR} = \gamma\mu_0\sqrt{H(H+M)}$. The minimum magnon frequency is $f_{min} = \gamma\mu_0 H$. The condition for the occurrence of three-magnon processes is $f_{FMR} > 2f_{min}$[7,8]. The above expressions yield the magnetic field range for the occurrence of three-magnon processes: $H < M/3$. Taking the example at 100 K, the magnetizations of GdIG and YIG is approximately 240 mT. Therefore, the influence of three-magnon processes is possible only if $\mu_0 H < 80$ mT. The resonant magnetic fields (~50-600 mT) in the FMR experiments mostly exceed 80 mT. Therefore, it can be inferred that three-magnon processes three-magnon and two-magnon processes do not hold significance in the ΔH versus *f*.

The effective Gilbert damping in the ferrimagnet GdIG is expressed as $\alpha_{eff0} \approx \frac{\alpha_{\mathrm{Fe}}L_{\mathrm{Fe}}+\alpha_{\mathrm{Gd}}L_{\mathrm{Gd}}}{|L_{\mathrm{Fe}}-L_{\mathrm{Gd}}|}$, where L_{Fe} (L_{Gd}) and α_{Fe} (α_{Gd}) represent the angular momentum and Gilbert damping of the Fe (Gd) sublattice, respectively. Under coherent spin pumping, the total damping is described as $\alpha_{tot} = \alpha_{eff0} + \Delta\alpha_1^{SP} \approx \frac{(\alpha_{\mathrm{Fe}}+\Delta\alpha_{Fe,1}^{SP})L_{\mathrm{Fe}}+(\alpha_{\mathrm{Gd}}+\Delta\alpha_{Gd,1}^{SP})L_{\mathrm{Gd}}}{|L_{\mathrm{Fe}}-L_{\mathrm{Gd}}|}$, where $\Delta\alpha_1^{SP}$ is the enhanced damping induced by spin pumping in GdIG. Besides, $\Delta\alpha_{\mathrm{Fe},1}^{SP}$ and $\Delta\alpha_{Gd,1}^{SP}$ are the enhanced damping of the effective Fe and Gd lattices due to coherent spin pumping. The magnetic moment of Gd mainly originates from the inner 4f shell-spin, which are more localized compared to the 3d shell-dominated moments in the Fe. The efficiency of spin pumping (or spin transfer) between 4f shell-spin and 3d shell-spin is much weaker than that of the Fe 3d shell-spin with itself or the conduction electrons. Therefore, the Fe lattices play a dominant role in the angular momentum transfer driven by coherent spin pumping. Neglecting the contribution from Gd in coherent spin pumping due to its weak efficiency, the enhanced damping $\Delta\alpha_1^{SP}$ driven by coherent spin pumping can be simplified as $\Delta\alpha_1^{SP} = \alpha_{tot} - \alpha_{eff0} \approx \frac{\Delta\alpha_{\mathrm{Fe},1}^{SP}}{|L_{\mathrm{Fe}}-L_{\mathrm{Gd}}|}$.

Based on both experimental data and fitted results shown in Fig. 2c in the main text, we evaluated the coupling region. As illustrated in Supplementary Fig. 7a, from a frequency

standpoint, the minimized separation of the H_r-f relations is ~13.4 GHz. Viewing it from a magnetic field perspective, as seen in Supplementary Fig. 7b, the minimum separation between the H_r-f relations is ~385 mT, and this corresponds to an anticrossing gap of ~ 0.5 GHz.

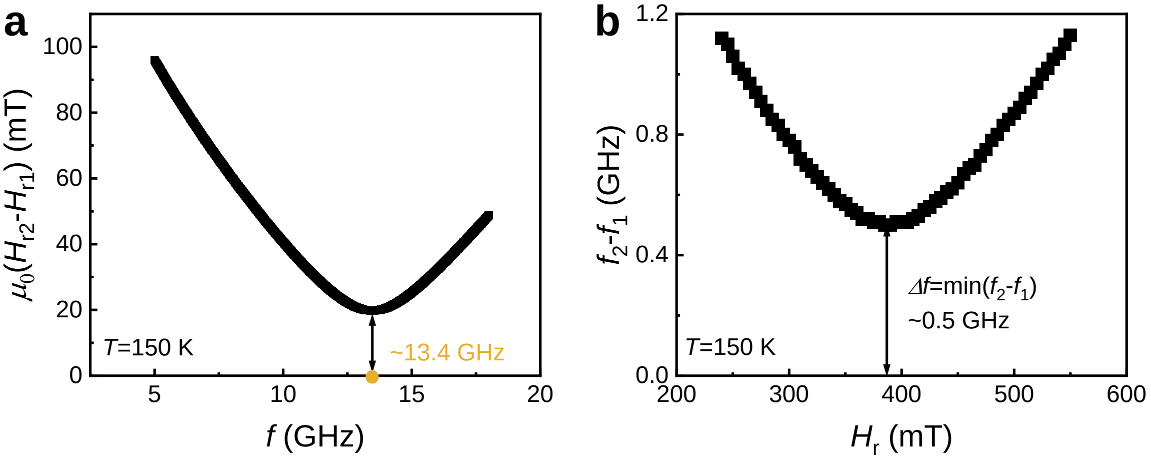

Supplementary Fig. 7| Separation of the two H_r-f relations (Fig. 2c in the main text). a, Separation of the two H_r-f relations from a frequency standpoint at 150 K. **b,** Separation of the two H_r-f relations from a magnetic field perspective at 150 K.

Vector Network Analyzer ferromagnetic resonance (VNA-FMR) measurements were also carried out by connecting VNA to a coplanar waveguide in a PPMS system. In the measurements, the frequency was scanned under various magnetic fields, while the transmission parameter S_{21} was recorded simultaneously. Given that the transmission signals are frequency and temperature dependent, the background-corrected field derivative of the measured transmission signals was conducted by $\frac{\partial S_{21}}{\partial H}$, yielding the VNA-FMR spectra[9,10]. As shown in Supplementary Fig. 8, these spectra highlight the anticrossings at 200 K and 150 K, along with variations in FMR absorption intensity due to magnon-magnon coupling. The magnon-magnon coupling strength is typically assessed by evaluating the minimal separation/anticrossing gap $(2g)$ in the frequency axis between the two modes around the anticrossing point. The system cooperativity is defined as $C = \frac{g^2}{\kappa_1 \kappa_2}$, where κ_1 and κ_2 represent the frequency linewidths of mode 1 and 2, respectively. As shown in Supplementary Fig. 8d, a distinct anticrossing gap indicates a strong magnon-magnon coupling with $2g \approx 0.50$ GHz and $C \approx 7$.

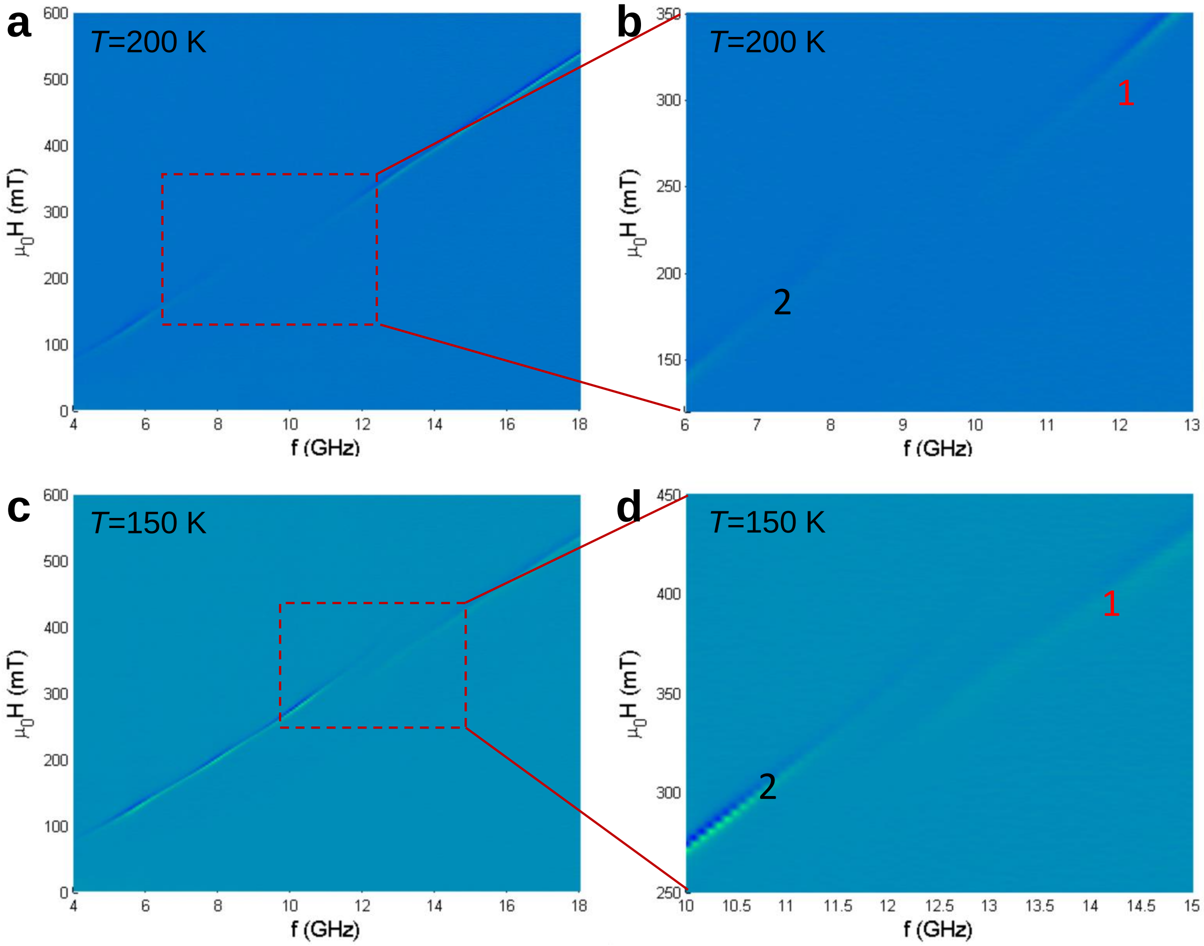

Supplementary Fig. 8| VNA-FMR spectra $\frac{\partial S_{21}}{\partial H}$ for GdIG/YIG as a function of magnetic field and frequency. a, VNA-FMR spectrum at 200 K. **b,** Enlargement of **a** around the anticrossing point. **c,** VNA-FMR spectrum at 150 K. **d,** Enlargement of **c** around the anticrossing point.

In spin pumping-driven ISHE experiments, the measured voltages typically comprise a combination of symmetric and antisymmetric Lorentzian terms. The symmetric term arises from spin pumping (V^{ISHE}), while the antisymmetric term is a result of the spin rectification effect (V_A). To extract the voltage signals from spin pumping, the following equation is employed:

$$V(H) = V_0 + V^{ISHE}\frac{\left(\frac{\Delta H}{2}\right)}{(H-H_r)^2+(\frac{\Delta H}{2})^2} + V^A\frac{\left(\frac{\Delta H}{2}\right)(H-H_r)}{(H-H_r)^2+(\frac{\Delta H}{2})^2} \quad \text{(S22)}$$

where V^{ISHE} and V^A are voltage amplitudes of symmetric and antisymmetric Lorentzian terms, respectively. V_0 is offset. Here, the extracted V_A is negligibly small due to the nearly absence of

the anisotropic magnetoresistance and anomalous Hall effects in insulator/Pt heterostructure (like YIG/Pt), as shown in Supplementary Figs. 9a. Supplementary Figs. 9b and c show the microwave power dependence of $V_{\rm ISHE}(H)$ at 300 K (above $T_{M,{\rm GdIG}}$) and 6 GHz for the resonant modes. The amplitudes $V_i^{\rm ISHE}$ of $V_{\rm ISHE}(H)$ for the mode i, (i=1, 2) were extracted using Eq. (S22). As shown in Supplementary Figs. 9d and e, both $V_1^{\rm ISHE}$ and $V_2^{\rm ISHE}$ are proportional to the power of microwave irradiation. Similar results can be obtained below $T_{M,{\rm GdIG}}$ (at 50 K), as shown in Supplementary Figs. 10a-e.

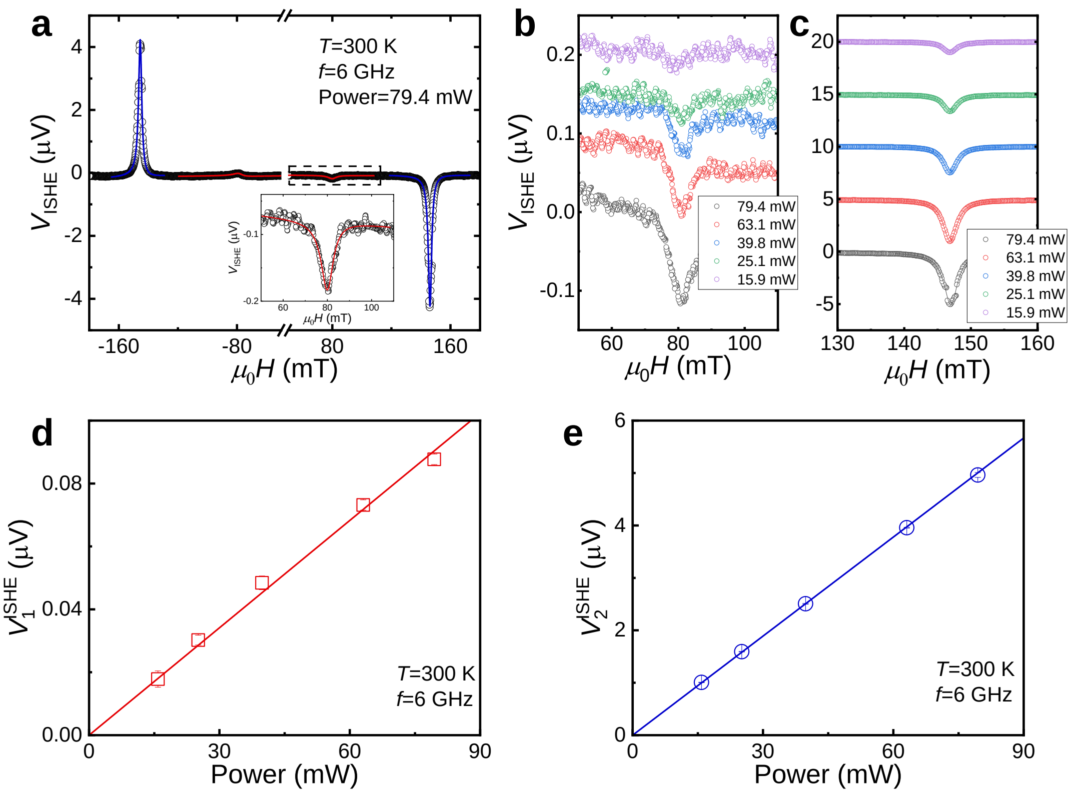

Supplementary Fig. 9| Spin pumping-driven ISHE measurements at 300 K. **a**, Measured ISHE voltage $V_{\rm ISHE}(H)$ as a function of magnetic field at f=6 GHz and T=300 K. **b-c**, Measured ISHE voltage $V_{\rm ISHE}(H)$ as a function of magnetic field corresponding to the mode 1 and mode 2 at f=6 GHz and T=300 K, at various microwave power, respectively. **d-e**, Extracted V_1^{ISHE} and V_2^{ISHE} as function of microwave power. Error bars represent fitting uncertainty.

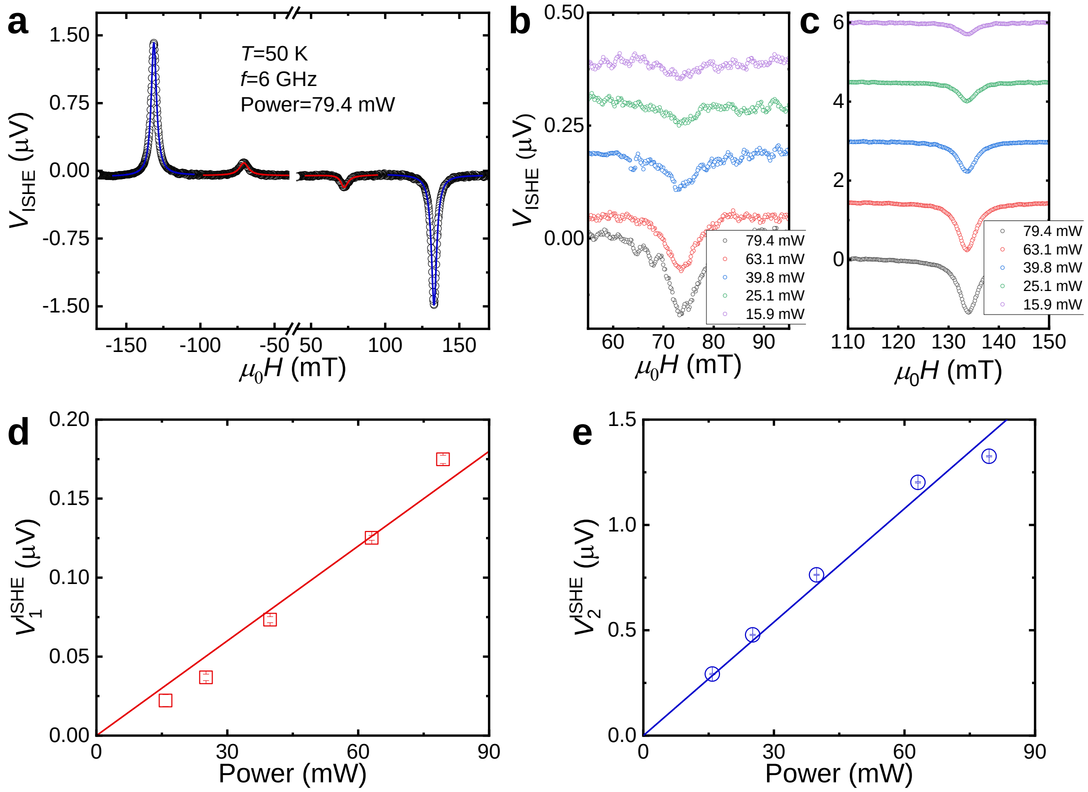

Supplementary Fig. 10| Spin pumping-driven ISHE measurements at 50 K. **a**, Measured ISHE voltage $V_{\mathrm{ISHE}}(H)$ as a function of magnetic field at f=6 GHz and T=50 K. **b-c**, Measured ISHE voltage $V_{\mathrm{ISHE}}(H)$ as a function of magnetic field corresponding to the mode 1 and mode 2 at f=6 GHz and T=300 K, at various microwave power, respectively. **d-e**, Extracted V_1^{ISHE} and V_2^{ISHE} as function of microwave power. Error bars represent fitting uncertainty.

The intensity of both FMR and spin pumping-induced ISHE signals can be approximatively as proportional to $\frac{t_i M_i}{\Delta H_i}$, where t_i and M_i are the thickness and magnetization of the magnetic i-layer, respectively. Therefore, at certain temperatures close to $T_{M,\mathrm{GdIG}}$, such as 150 K and 200 K, the FMR peak with a small amplitude is exceptionally weak, with a small amplitude that makes it almost indiscernible. Moreover, the mode hybridization further enables the transfer of energy between magnon excitations, as shown in Supplementary Fig. 11a. The corresponding ISHE voltages may also be indiscernible due to the weak FMR signals, as shown in Supplementary Fig. 11b.

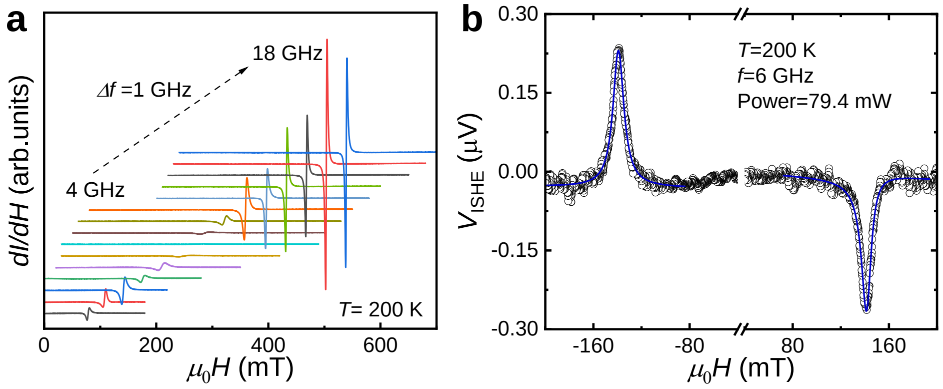

Supplementary Fig. 11| FMR and spin pumping-induced ISHE measurements at 200 K. a, FMR spectra measured at various frequencies and *T*=300 K. **b**, Measured ISHE voltage $V_{\rm ISHE}(H)$ as a function of magnetic field at *f*=6 GHz.

To characterize the difference in magnon transport between the two hybrid modes, we define a ratio ξ as $\xi = \frac{V_1^{ISHE}}{P_1'} / \frac{V_2^{ISHE}}{P_2'}$, where P_1' and P_2' represent the amplitudes of the two resonance peaks at low and high magnetic fields, respectively, as shown in Supplementary Fig. 12a. Supplementary Fig. 12b exhibits $V_{\rm ISHE}(H)$ as a function of magnetic field at RF power of 79.4 mW, a fixed frequency of 6 GHz and *T*=100 K. It is evident that that V_1^{ISHE} is very weak due to large damping, small net moments in the GdIG layer, and magnon hybridization between the YIG and GdIG layers. Supplementary Fig. 12c shows the frequency dependent ξ at various temperatures. Note that the values of ξ are greater than 1 above $T_{M,\rm GdIG}$ while less than 1 below $T_{M,\rm GdIG}$ at all measured frequency points.

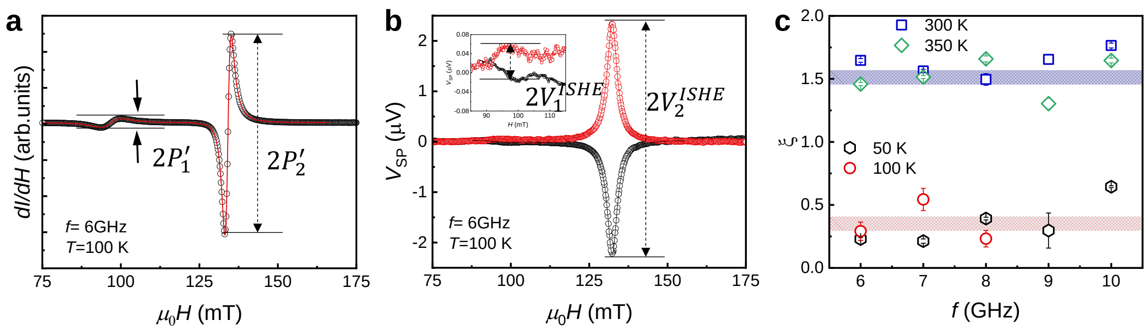

Supplementary Fig. 12| Spin pumping-driven ISHE measurements. **a**, Derivative FMR absorption at *f*=6 GHz and *T*=100 K. **b**, Measured ISHE voltage $V_{\rm ISHE}(H)$ as a function of magnetic field at a microwave power of 79.4 mW, a fixed frequency of 6 GHz and *T*=100 K. The black and red circles are the ISHE voltage signals acquired with sweeping positive and negative

magnetic fields, respectively. The inset is the zoomed-in view of the hybrid mode 1. **c**, Frequency dependent ξ at various temperatures. Error bars represent fitting uncertainty.

Supplementary Note 5: Spin Seebeck effect experiments

Spin Seebeck effect measurements are performed, as schematized in Supplementary Fig. 13a. An insulating SiO_2 layer was heated by a Ti/Pt strip with a charge current of 10 mA, giving rise to a temperature gradient (∇T) along the z-axis. The incoherent spin waves induced by the ∇T via the SSE in GdIG/YIG carry spin angular momentum into the adjacent Pt layer. The voltage signals (V_{SSE}) were detected in the Pt layer via ISHE by sweeping the applied magnetic field along the y-axis. The SSE measurement is a bulk-sensitive magnetometry technique to assess the magnetic configuration. As shown in Supplementary Fig. 13b, the V_{SSE} becomes saturated when the applied magnetic field surpasses ~50 mT. These support the presence of a stable magnetic configuration within the magnetic field range of ~50-600 mT, which corresponds to the range employed in our FMR measurements.

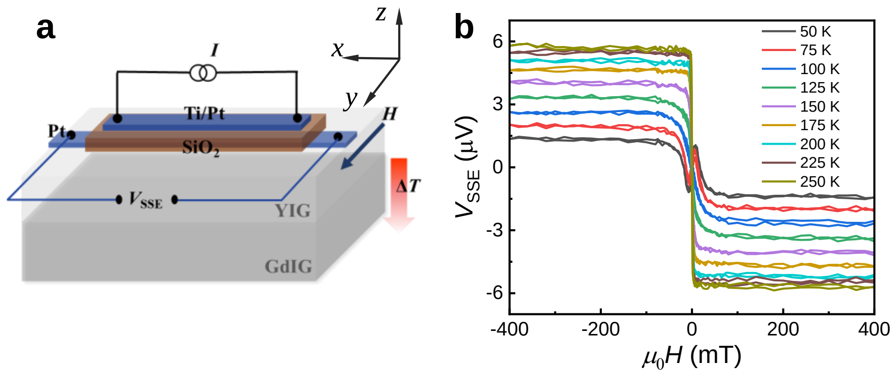

Supplementary Fig. 13 Spin Seebeck effect-driven ISHE measurements. a, Schematic of the SSE measurements for GdIG/YIG. **b,** Measured SSE signals (V_{SSE}) as a function of the applied magnetic field at various temperatures.

References

1 Harris, A. B. Spin-Wave Spectra of Yttrium and Gadolinium Iron Garnet. *Phys. Rev.* **132**, 2398 (1963).

2 Li, S., Shen, K. & Xia, K. Magnon hybridization in ferrimagnetic heterostructures. *Phys. Rev. B* **102**, 224413 (2020).

3 Shen, K. Temperature-switched anomaly in the spin Seebeck effect in Gd3Fe5O12. *Phys. Rev. B* **99**, 024417 (2019).

4 Shen, K. Magnon spin transport around the compensation magnetic field in easy-plane antiferromagnetic insulators. *J. Appl. Phys.* **129**, 223906 (2021).

5 Heinrich, B. *et al.* Dynamic exchange coupling in magnetic bilayers. *Phys. Rev. Lett.* **90**, 187601 (2003).
6 Li, Y. *et al.* Coherent Spin Pumping in a Strongly Coupled Magnon-Magnon Hybrid System. *Phys. Rev. Lett.* **124**, 117202 (2020).
7 Kurebayashi, H. *et al.* Controlled enhancement of spin-current emission by three-magnon splitting. *Nat. Mater.* **10**, 660-664 (2011).
8 Lee, O. *et al.* Nonlinear Magnon Polaritons. *Phys. Rev. Lett.* **130**, 046703 (2023).
9 Maier-Flaig, H. *et al.* Note: Derivative divide, a method for the analysis of broadband ferromagnetic resonance in the frequency domain. *Rev. Sci. Instrum.* **89**, 076101 (2018).
10 Klingler, S. *et al.* Spin-Torque Excitation of Perpendicular Standing Spin Waves in Coupled YIG/Co Heterostructures. *Phys. Rev. Lett.* **120**, 127201 (2018).
